# History of the Inoculation for the Cow-Pox

**Published:** 1799-05

**Authors:** 


### *Medical and Physical Journal.*

VOL. I.] MAY, 1799. [NO. III.

### *History of the Inoculation for the Cow-pox,*

[Continued from page 120 of our last.]

THE annexed plate requires no other explanation than what is contained in the relation of the case which refers to it; page 118 to 120 of No. II.

As we had wished to lay before the public an impartial, unadorned case of cow-pox, just as it might impress an anxious and attentive parent, and just as described by him, from notes made during its daily progress; we think ourselves peculiarly happy in having all the desiderata united in one individual instance. The plain and simple description and relation of a person unconnected with the profession of medicine, must be far more satisfactory to the public, than any case selected for the occasion by a practitioner, who might wish to recommend the new disease.

The appearance, and degree of severity of the cow-pox, when produced by inoculation, agrees with Mr. WALKER's account, in about 97 cases in 100, of those inoculated in London and its neighbourhood. In a very few instances, however, during the last six weeks, an *eruption* has taken place over the whole body, which, by a cursory observer, might have been mistaken for the small-pox. We have seen some of these cases; the *first* of which was inoculated with matter taken from the cow; the *second* with matter taken from the first. Each of these children had a numerous eruption, but were in no danger. The *third* and *fourth* were inoculated with matter taken from the second, and had an eruption, but much slighter than the first or second. If it ever should become a question, which is not improbable, whether the cow-pox, by repetition in the human subject, may not degenerate into the small-pox, it may then be necessary to point out the distinction which subsisted between the two diseases, when the former was first introduced as a preventative of the latter. At present we shall only add, that Dr. JENNER's second Volume has been expected every day of the last week; and that a work on this subject by Dr. WOODVILLE, who appears to have inoculated twenty times as many patients as any other practitioner, will appear in the first week of May.

T. B.

*April 28.*

